# A new approach for the pixel map sensitivity (PMS) evaluation of an electronic portal imaging device (EPID)

**DOI:** 10.1120/jacmp.v14i6.4420

**Published:** 2013-11-04

**Authors:** Alberto Boriano, Francesco Lucio, Elisa Calamia, Elvio Russi, Flavio Marchetto

**Affiliations:** ^1^ Radiotherapy Department S. Croce e Carle Hospital Cuneo Italy; ^2^ INFN Sezione di Torino Torino Italy

**Keywords:** EPID, calibration, portal dosimetry, dose verification

## Abstract

When using an electronic portal imaging device (EPID) for dosimetric verifications, the calibration of the sensitive area is of paramount importance. Two calibration methods are generally adopted: one, empirical, based on an external reference dosimeter or on multiple narrow beam irradiations, and one based on the EPID response simulation. In this paper we present an alternative approach based on an intercalibration procedure, independent from external dosimeters and from simulations, and is quick and easy to perform. Each element of a detector matrix is characterized by a different gain; the aim of the calibration procedure is to relate the gain of each element to a reference one. The method that we used to compute the relative gains is based on recursive acquisitions with the EPID placed in different positions, assuming a constant fluence of the beam for subsequent deliveries. By applying an established procedure and analysis algorithm, the EPID calibration was repeated in several working conditions. Data show that both the photons energy and the presence of a medium between the source and the detector affect the calibration coefficients less than 1%. The calibration coefficients were then applied to the acquired images, comparing the EPID dose images with films. Measurements were performed with open field, placing the film at the level of the EPID. The standard deviation of the distribution of the point‐to‐point difference is 0.6%. An approach of this type for the EPID calibration has many advantages with respect to the standard methods — it does not need an external dosimeter, it is not related to the irradiation techniques, and it is easy to implement in the clinical practice. Moreover, it can be applied in case of transit or nontransit dosimetry, solving the problem of the EPID calibration independently from the dose reconstruction method.

PACS number: 87.56.‐v

## I. INTRODUCTION

The introduction of several intensity modulation techniques in external beam radiotherapy has led, in the last two decades, to an increase in the required accuracy for the dose delivered to the patients. Pretreatment verifications are normally performed in IMRT treatment plans, with the more general aim of an *in vivo* dose verification. On the other side, the increase in the number of patients treated with these kinds of techniques requires verification tools (in phantom or *in vivo*) that are fast and accurate.

Electronic portal imaging device (EPID), developed for the patient positioning verification, has been and still is investigated for dosimetric application. Over the past 20 years the number of publications on EPID has considerably increased (one of the first papers was presented by van Herk^(1)^ in 1991), driven by EPID's many advantages: high spatial resolution, fast image acquisition, and digital format. Additionally, for a number of years, several commercial softwares that allow a dosimetric use of the EPID are available on the market.^2^, ^3^, ^4^

In a literature review, van Elmpt et al.^(5)^ defined EPID dosimetry as “determination of the dose in the detector, patient, or phantom, or determination of the incident energy fluence” based on measurement without (nontransit dosimetry) or with (transit dosimetry) an attenuation medium between the source and the detector.

Independently from the dosimetry method, the first step in using the EPID as a detector for dose measurements is the definition of a dedicated calibration that leads to the evaluation of the pixel sensitivity map (PSM). In this paper we intend to apply PSM on top of the calibration coefficients determined with the flood‐field procedure. For clarity sake, we remark that the flood‐field is an image acquired with a large open field. The pixel‐to‐pixel response differences can be corrected by division of raw images by the flood‐field calibration image; the beam profile is present in both the raw image and FF image, and is therefore washed out of the final stored image. Thus, the PSM corrects for the open field disuniformity restoring the (real) beam profile.

Due to the oversensitivity to low energy,^6^, ^7^, ^8^, ^9^, ^10^ the relation between sensitivity and dose depends on the off‐axis position of a specific pixel, as well as the thickness of the phantom or of the patient in the beam. Once the EPID is calibrated (i.e., the PSM is known), the final dose computation must include the oversensitivity correction. A considerable amount of papers describe procedures suitable to verify a point dose, a 2D dose matrix at EPID or patient level, or a 3D matrix, based both on nontransmission and transmission methods.^(5)^

For the evaluation of the PSM, two methods are generally used: simulation of response of the panel as a function of the incident radiation (i.e., simulation of the gray scale pixel value) or empirical conversion of the pixel count (the gray scale value) to a dose value. The first approach models the detector response applying, normally, a Monte Carlo simulation.^(^ ^6^ ^,^ ^11^, ^12^, ^13^ ^)^ This method can be a useful tool to derive, test, and validate assumptions concerning the dose response of the EPID. The main drawback is that detailed information of the technical design of the EPID is not always available and long calculation time is needed.

The second calibration approach applies (semi) empirical models to convert the measured grey‐scale image of EPID into a portal dose image.^14^, ^15^, ^16^, ^17^ An approach of this type, widely described in literature, is based on the conversion to a relative or to an absolute dose by using a calibrated detector, usually an ionization chamber inside either a water tank, either a miniphantom or a film.^(^ ^18^ ^,^ ^19^ ^)^ The resulting dose map can be directly compared to a conventional dosimeter measurement, which is an obvious advantage of the method. This conversion method is simpler and faster than a Monte Carlo approach, and therefore more suited for implementation in clinical routine. The drawback is that these calibration models can be too simple to cover all treatment techniques and irradiation configurations.®

An alternative empirical method for the evaluation of the PSM, based on multiple acquisitions, was presented by Greer in 2006.^(20)^ Moving the detector in the cross‐plane direction, the author derived the pixel sensitivity for the central axis profile in the cross‐plane direction. The two‐dimensional PSM was then obtained from the radial extension of the ratio of the cross‐plane central axis profile of an open field to the cross‐plane central axis profile of the pixel sensitivity variation. As described by the author, this procedure is affected by few limitations: mainly, the assumption of radial symmetry of the ratio between the two profiles, and the assumption of flatness of the field used for the measurements.

Assuming that the PSM (as expressed by Greer) is the response of all the sensors when irradiated with the same fluence, in this paper we present a new approach for the evaluation of the sensitivity of an EPID using an intercalibration procedure. In particular, the response of each element is expressed in terms of gain relative to the response of pixels chosen as reference.

The first and more general method based on this idea was patented by Simon et al.^(21)^ in 2000 and again by Simon et al.^(22)^ a decade later, carefully analyzed the outcome of the algorithm as a function of the output stability of the accelerator. In a following work, Donetti et al.^(23)^ used the same principle to calibrate a square matrix of N×N detectors, applying it to a matrix of 32×32 ionization chambers. Since these methods require a detector rotation, as described by Simon and Donetti they cannot be included among the EPID PMS evaluation methods. Taking into account the moving capability of our EPID, we have developed a similar method, computing (and comparing) PSMs of the detector in different setups. A first approach of this type, base on two shifts, was briefly presented as a poster by Wang et al. in 2012 at ASTRO 2012.^(24)^

In this paper we describe the procedure, the algorithm, the setups, and the results of calibration, assuming the film as reference detector. We use the words sensitivity, gain, and response as synonymous.

## II. MATERIALS AND METHODS

### A. Accelerator and EPID

All measurements described in this paper have been performed using an Elekta SL15I accelerator, with nominal photons energies of 6, 10, and 15 MV (Elekta, Crawley, UK). The accelerator was equipped with a multileaf collimator consisting of 40 leaf pairs, with a projected leaf width of 1 cm at the isocenter. The EPID was an Elekta iViewGT, built with the following layers: aluminum top cover, air gap, copper plate, graphite layer, scintillator plate Gadox (terbium‐doped gadolinium), and attenuating film. The detector is a PerkinElmer Amorphous Silicon (a‐Si) panel (PerkinElmer, Waltham, MA) that provides a resolution of 1024×1024 16‐bit pixel. The source to detector distance is fixed and is ~157cm. Each pixel has a pitch of 0.400 mm and the resulting sensitive area is $\sim 41\times 41\;{\text{cm}}^{2}$. Projected to the isocenter, the sensitive area is $\sim 25\times 25\;{\text{cm}}^{2}$ with a pixel resolution of 0.25 mm.^25^, ^26^, ^27^

For images acquisition, the iViewGT software, version 3.4 (Elekta), has been used. Frame averaging has been set to the maximum and frames have been accumulated for the complete beam. Three calibration matrixes have been applied to the acquired images: the flood field (FF) correction from a single level calibration, the gain correction, and the bad pixel correction. The offset (or dark‐field (DF)) correction automatically started after five frames without beam. These corrections are applied in the standard image acquisition procedure. Images have been acquired with the original resolution (0.25 mm at the isocenter plane), exported in tiff format and grouped to obtain 5 mm pixel side (always at the isocenter plane). Throughout the paper we refer as pixel to the grouped ensemble rather than to a single one. Indeed the extension of the pixel has been decided balancing two opposite effects: the systematic error which, due to the propagation, tends to increase with the number of pixels, and the sensitivity of the measurement which conversely decreases by increasing the pixel dimension. Moreover, this algorithm requires that the pixel dimension and the EPID shift coincide. Finally, a pixel dimension of 5 mm was found to be the best compromise. Our calibration procedure needs five consecutive acquisitions. Because the absolute counts of each acquisition is of paramount importance, in the export phase the five images were not normalized (images are acquired consecutively as part of a five‐segment IMRT field, setting the variable “ImrtDosimetricWeighting” = 1 in the sri.ini file).

### B. Algorithms

#### B.1 Calibration procedure

The basic principle of an intercalibration procedure can be summarized as follows: the relative gain of the sensors of a 2D matrix can be evaluated with a sequence of irradiations with the beam which extends over the full detector. To allow recursive multiple comparisons, at each beam delivery the relative position of the detector with respect to the beam has to be shifted. At each step of the calibration sequence, a given spot of the beam open field is eyed by a different detector pixel, thus allowing the computation of the relative gain between several pixels. The main assumption is that the overall integrated fluence and the shape of the beam are constant for the successive deliveries.

The movements allowed to the EPID are in the gantry table and left‐right directions.

The calibration procedure required five shifts or acquisitions, each performed with the detector at fixed displacement and with a beam open field. The first acquisition was done with the EPID in the central position with respect to the beam. The other four irradiations were done with the EPID displaced sideways and up‐down, symmetrically with respect to the central position. Each shift was equal to the pixel width. In this paper, two different coordinate systems are used: one, (x, y), is linked to the accelerator, the other, (i, j), linked to the EPID. The x‐axis coincides with the gantry rotation axis and the y‐axis is parallel to the direction gantry table. Both the x‐ and y‐axis lay on the EPID plane. The EPID coordinates, (i, j), represent the corresponding pixel and are aligned with x and γ respectively. The center (x,y)=(0,0) is located at the intersection between the plane defined by the EPID plane and the projection of the isocenter perpendicular to it.

During the five acquisitions of the calibration procedure, the center of the EPID has been placed respectively at the following (x, y) coordinates: (0,0), (0,1), (0,‐1), (1,0), (‐1,0).

Let us introduce the following definitions:

- With “A” we identify the measurement (or acquisition) taken with the detector at the central position and with “B”, a shifted one
- F(i, j) is the fluence delivered to the pixel (i, j) area
- C(i, j) are the counts proportional to the collected charge on the pixel (i, j)

G(i, j) is the calibration coefficient (or factor), unknown, of the pixel (i, j). In the present paper, we give to G(i, j) the meaning of the PSM. In this context, fluence and counts are related by the equation:

(1) F(i,j)=C(i,j)×G(i,j)

Considering a pixel (i, j) and the EPID in the position A, Eq. (1) can be written as:

(2) $$F_{A}(i,j)=C_{A}(i,j)\times G(i,j)$$

If we assume that in position B the pixel (i, j) is replaced by the pixel (p, q), Eq. (2) becomes:

(3) $$F_{B}(p,q)=C_{B}(p,q)\times G(p,q)$$

The assumption that the beam does not change shape and fluence for the subsequent deliveries is expressed by the equation $F_{A}(i,\;j)=F_{B}(p,\;q)$. One can then write that:

(4(a)) $$C_{A}(i,j)\times G(i,j)=C_{B}(p,q)\times G(p,q)$$

(4(b)) $$G(p,q)=\frac{C_{A}(i,j)}{C_{B}(p,q)}\times G(i,j)$$

Equation (4b) shows the aim of a general intercalibration procedure: the calibration coefficient of each sensor can be related to the coefficient of a sensor chosen as reference.

#### B.2 Calibration algorithms

**Figure 1:**
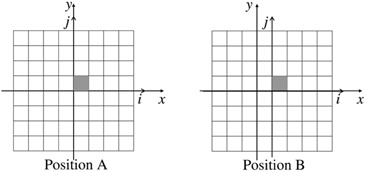
Sketch of two steps of calibrations: irradiation A, the center of the EPID at the coordinates (x,y)=(0,0), and irradiation B, at (x,y)=(1,0), respectively. The gray pixel is the reference pixel and its coordinates (linked to the EPID (i,j) system) are in both A and B configurations (i,j)=(1,1).

We describe now a particular case consisting in two consecutive irradiations (see Fig. 1):

1. irradiation A: the EPID matrix is centered with respect to the beam;
2. irradiation B: the EPID matrix is shifted by one pixel in the cross direction towards positive x. As mentioned above, the reference system (x,y) is linked to the accelerator, while (i,j) is linked to the EPID matrix and identifies the positions to which the pixels refers.

In Fig. 1 irradiation A, the gray pixel, that we name as (1,1), is located in position (1,1), while in irradiation B, the same pixel has been displaced to (2,1). Furthermore we consider pixel (1,1) as reference and we arbitrarily fixed G(1,1)=1. The aim of the procedure is to relate all the coefficients to G(1,1).

Following the sketch in Fig. 1 and assuming the beam fluence F being fixed, one can write the equation:

(5) $$F_{A}(2,1)=F_{B}(1,1)$$

Replacing the indexes (i,j) and (p,q) with (1,1) and (2,1), Eq. (4b) can be written as:

(6) $$G(2,1)=\frac{C_{B}(1,1)\times G(1,1)}{C_{A}(2,1)}$$

Adopting the same procedure for another pair of points which occupy the same beam spot, displacing the EPID from A to B, for example pixel (2,1) and pixel (3,1), and by applying recursively Eq. (6), one can relate pixel (3,1) to pixel (1,1):

(7) $$G(3,1)=\frac{C_{B}(2,1)\times G(2,1)}{C_{A}(3,1)}=\frac{C_{B}(2,1)\times C_{B}(1,1)}{C_{A}(3,1)\times C_{A}(2,1)}\times G(1,1)\;\;\;$$

Assuming a matrix of N×N pixels and defining M=N/2, the generic factor $G_{B}(i,1)$ (where the subscript *B* is relative to the EPID position and *i* is in the range (2, M)) can be written as:

(8a) $$G(3,1)=\frac{C_{B}(2,1)\times G(2,1)}{C_{A}(3,1)}=\frac{C_{B}(2,1)\times C_{B}(1,1)}{C_{A}(3,1)\times C_{A}(2,1)}\times G(1,1)\;\;\;\text{for}\;i\;\varepsilon (2,M)$$

Following the same procedure, but stepping in the opposite direction, Eq. (5) becomes:

(9) $$F_{A}(1,1)=F_{B}(-1,1)$$

The generic coefficient G(i,1) with i in the range (−M,−1), is given by:

(8b) $$G_{B}(i,1)=\frac{\prod_{k=-1}^{-i-1}C_{A}(-k,1)}{\prod_{k=1}^{-i}C_{B}(-k,1)}\times G(1,1)\;\;\;\;\;\;\text{for}\;\;\;i\;\;\varepsilon \;(‐M,‐1)$$

Equations 9, 10 allow to determine the calibration coefficient for all the pixels in the line j=1, relatively to pixel (1,1). Obviously, applying the same procedure, one can extend the method to any other line, thus computing the calibration coefficient of each pixel (i,j) as a function of the pixel (1,j).

**Figure 2:**
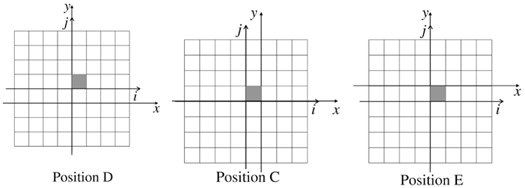
Sketch of the last three steps of calibrations EPID in position (x,y)=(0,1),(x,y)=(−1,0)and(x,y)=(0,−1).

Let us now consider a new measurement setup, namely the one shown in Fig. 2 — irradiation D where the EPID has been displaced along the gantry table direction.

Comparing irradiation A to irradiation D, Eq. (5) can be rewritten as:

(9) $$F_{A}(1,2)=F_{D}(1,1)$$

The calibration coefficients for pixels in the ranges (2,M) and (‐M,‐1) can be formulated as:

(10(a)) $$G_{D}(i,j)=\frac{\prod_{k=1}^{j-1}C_{D}(1,k)}{\prod_{k=2}^{j}C_{A}(1,k)}\times G(1,1)\;\;\;\;\;\;\text{for}\;\;\;j\;\;\varepsilon \;(2,M)$$

(10(b)) $$G_{D}(i,j)=\frac{\prod_{k=-1}^{-j-1}C_{A}(1,-k)}{\prod_{k=1}^{-j}C_{D}(1,-k)}\times G(1,1)\;\;\;\;\;\;\text{for}\;\;\;j\;\;\varepsilon \;(‐M,‐1)$$

Finally, we can consider the last two EPID positions, one on the left‐right negative X direction (Fig. 2, irradiation C), the other one on the gantry table positive Y direction (Fig. 2, irradiation E).

Using as reference the acquisition with the EPID in the position A, the positions C and E lead, respectively, to a calibration matrix $G_{C}(i,1)$ and $G_{E}(1,j)$.

Summarizing, to calibrate the pixels of the central row, j=1, as a function of G(1,1) are needed two acquisitions with configuration as Fig. 1 irradiation A and B, respectively. Indeed the above procedure gives enough constraints to calibrate the pixels on any given row j as a function of the pixel (1,j).

Generalizing, the generic row j=n can be calibrated as a function of pixel (1,n), like the generic column i=m can be calibrated in function of pixel (m,1). Once, for instance, the row j=1 has been calibrated, the response, relative to the pixel (1,1), of the generic pixel (m,1) is known. It is thus possible to use it to recalibrate the generic column i=m, and, obviously, vice versa. Assuming the acquisitions setup to be the positions A, B, and D, it is possible to describe the path to calculate the generic coefficient G(i,j) as follows. Equations 9, 10 can be written in a simplified form as:

(11) $$G_{B}(i,1)=f_{B,i}(G(1,1))$$

In the same way, 13, 14 can be written as:

(12) $$G_{D}(m,j)=f_{D,j}(G(m,1))$$

Substituting Eq. (11) into the Eq. (12), it is possible to compute a global calibration using, in order, steps B and D. The generic coefficient $G_{B,D}$ is:

(13) $$G_{B,D}(m,j)=f_{D,j}(G(m,1))=f_{D,j}[f_{B,i}(G(1,1))]$$

The vice versa will lead to a different set of calibration coefficients:

(14) $$G_{D,B}(i,n)=f_{B,i}(G(1,n))=f_{B,i}[f_{D,i}(G(1,1))]$$

Three acquisitions (one central, one in the left‐right direction, and one in the gantry table direction) could be sufficient to compute the whole calibration matrix. However, to reduce (and to evaluate) the errors, we decided to use the five measurement steps described above. The number of global calibration sets obtained is, in this case, eight: $G_{B,D},\;G_{D,B},\;G_{B,E},\;G_{E,B},\;G_{C,D},\;G_{D,c},\;G_{C,E},\;G_{E,C}$. The single pixel coefficient is thus computed as average over the eight values, while its spread gives the uncertainty of the calibration.

#### B.3 Output and symmetry variation corrections

The entire procedure relies on the repetition of the beam for all the steps of the procedure both for what concerns overall fluence and shape. Conversely, any beam deviation may influence the results. We categorize the beam variation as coming from two possible classes of perturbation: a) output variation, which represents the overall beam fluence deviation going from one irradiation to the next, and b) symmetry variation, which accounts for the beam shape change. The output variation can be due to the nonperfect linearity of the monitor chambers, whereas the symmetry variation can be ascribed to even a small lateral displacement of the beam.

Since the computed gain of a generic pixel, n, is a function of the counts of the pixels in the range [1; n‐1], an irradiation field variation can lead to a systematic (in case of output variation) or quasisystematic (in case of symmetry variation) error propagation. Consequently, a method that corrects for the interacquisitions variations has been introduced.

#### B.3.1 Output variation

As described above, we define as “A” the central irradiation and as “C” the shifted one of Fig. 2. We consider now only the central cross‐plane profile (i.e., the profile that lies on the shifting direction), excluding the external pixels of the profile. With regard to the irradiation “A” and using the notation described above, the pixels taken in account have thus coordinate (i,1), with i in a range [−k,k]. We fixed k=(N/2−3), excluding the external three pixels (we note that N is the total number of pixels in the profile). If the shift amount is equal to the pixel dimension, one can assert that:

(15) $$\prod_{i=-k}^{k}C_{A}(i,1)\cdot G(i,1)=\prod_{i=-(k-1)}^{k+1}C_{C}(i,1)\cdot G(i,1)$$

Since the calibration coefficients G(i,j) are relative to a flood field calibrated image (i.e., the G(i,j) are nearly symmetric with respect to the center of the beam), it's reasonable to assume that G(−k,1)~G(k+1,1). Moreover, one can reasonably assume that the gains do not change going from one acquisition to the next one. Under these assumptions, Eq. (15) simplifies as follows:

(16) $$\prod_{i=-k}^{k}C_{A}(i,1)=\prod_{i=-(k-1)}^{k+1}C_{C}(i,1)$$

In fact, Eq. (16) stands true if there are no beam output variations. To allow a fluctuation of the beam fluence, one has to introduce a correction factor f to account for the impact on the counting of each pixel:

(17) $$\prod_{i=-k}^{k}C_{A}(i,1)=\prod_{i=-(k-1)}^{k+1}f^{2k}C_{C}(i,1)$$

Thus one can derive the correction factor f from the following Eq.:

(18) $$f={\left(\frac{\prod_{i=-k}^{k}C_{A}(i,1)}{\prod_{i=-(k-1)}^{k+1}C_{C}(i,1)}\right)}^{\frac{1}{2k}}$$

Finally f is applied to each pixel count of “C” to correct them and account for the output variation of “C” with respect to “A”. The same algorithm is applied to normalize “B”, “D”, and “E” with respect to “A”.

#### B.3.2 Symmetry variation

Analyzing the profiles of different subsequent EPID acquisitions, occasionally we found beam symmetry variations. In fact, whereas the beam profile is very stable in the left‐right direction, in our system we observed a random variation in the gantry table direction up to ±0.15%.

Following the parameterization suggested by Simon et al.,^(22)^ we describe the shape of the profile variation with the approximated formula, as reported in Eq. (19):

(19) $$pert(y)=u\cdot \text{sin}\left(\pi \cdot \frac{y}{length}\right)$$

where *pert* means perturbation, *y* is the position with respect to the center, *length* is the length of the profile, and *u* is the maximum beam variation, while the sign of *u* (positive or negative) defines the slope of the asymmetry.

To cure the beam symmetry variation within our procedure, we elaborated an iterative method to reduce the errors induced by the anomalous behavior of the beam.

The method starts from the fact that the final PMS is calculated as the average value over eight different PMSs computed with one central, “A”, and four shifted acquisitions, namely “B”, “C”, “D”, and “E”. Thus, if we assume that one out of four shifted acquisitions has a symmetry variation, the induced errors are mitigated by the other acquisitions over the final PMS.

Moreover, once the final (average) PMS has been calculated, the same is applied to the five acquired images. Then, the differences between the central and each one of the four shifted calibrated images are computed. Ideally, provided that no symmetry variation occurred during the acquisitions, all the four differences should be zero. On the other hand, a symmetry variation along any direction, gantry table or left‐right, induces a difference profile that can be described by Eq. (19). A fit of the difference profile to Eq. (19) is then performed (in our particular case, Elekta SL15I but only in the gantry table direction) to extract the u parameter, which represents the amplitude of the sine function. The derived function is applied to correct the original shifted image.

Finally, the PMS is recomputed with corrected shifted images.

#### B.4 Dose image

The open field, known in literature as flood‐field (FF), is normally used to determine the individual pixel sensitivity variation. The basic assumption is that the open field provides a flat dose distribution over the EPID area. To correct for the deviation from flatness of the flood‐field, we apply the above described calibration procedure, as follows.

The raw image acquired with a beam profile is ${\text{IM}}^{\text{raw}}$, and defining as dark field (DF) the image acquired without radiation, ${\text{FF}}_{\text{mean}}$ the mean value of the FF image, the postprocessed FF‐corrected image ${\text{IM}}^{\text{EPID}}$ computed by the EPID software is defined as:

(20) $$IM_{ij}^{EPID}=\left(\frac{IM_{ij}^{raw}-DF_{ij}}{FF_{ij}}\right)\times FF_{mean}$$

where the indices *i* and *j* identify a pixel on the EPID image.

${\text{IM}}^{\text{EPID}}$ contains the flat field approximation that can be corrected for by applying the correction coefficients G.

The final dose image $D^{\text{EPID}}$ is thus obtained by the relation:

(21) $$D_{ij}^{EPID}=IM_{ij}^{EPID}\times G_{ij}\times c$$

where *c* is a constant value dependent from setup condition that can be used to convert from EPID read out units to dose. In this analysis, c has been set to unity, giving to the dose image the meaning of a relative dose.

As already mentioned in the Introduction, the dose image is the response of EPID to the incident radiation. This means that, especially due to the overresponse of EPID to low‐energy photons, Eq. (21) is the first order of image correction in any dosimetric process. The second one is function of the method of dose reconstruction and, mainly, should correct for the lateral scatter (e.g., off‐axis, output factor, scatter in phantom).

### C. Measurements

Two sets of measurements were done: the first one to compute the calibration matrix ($G_{\text{ij}}$) and to check the goodness of the procedure. The second, following Eq. (16), to verify the dose reconstruction.

#### C.1 Calibration setup

To avoid the irradiation of the EPID read out system (that would leads to images artifacts and damage to the electronics), also considering the shift of the detector, collimator jaws were set to $23\times 23\;{\text{cm}}^{2}$, delivering 30 MU for each field at, approximately, 300MU/min.

Photon energy and scatter dependence were evaluated performing the calibration process in different setups. For the energy dependence, a 4 cm thick slab phantom was placed over the treatment couch, for a SSD of 98 cm, and two different PSMs were determined: one with photon energy of 6 MV, the other of 15 MV. The phantom thickness was set to 4 cm in order to minimize the modification of the photons energies spectrum and, at the same time, have a scatter component.

Similarly, the scatter dependence was evaluated from the comparison of two PSMs measured in a minimum (no phantom between the EPID and the source) and maximum (20 cm thick slab phantom, SSD of 90 cm) scatter condition. In this case the photon energy was fixed to 6 MV.

Acquisition parameters of the iViewGT have been already described in Materials and Methods section A above.

#### C.2 Test of the calibration procedure

As described above, this kind of procedure gives as result eight sets of calibration coefficients; for each pixel of the detector the mean value and the standard deviation over the eight coefficients were computed. As first indicator of the goodness of the procedure, we used the distribution of the standard deviations and the distribution of the residuals (residual is defined as difference among the single pixel mean value and, relative to that pixel, the value of each one of the eight calibration coefficients). Even if the spread of these two distributions does not give information about the matching between the measured and the real absorbed dose, it can quantify the consistency of the procedure. In fact the residuals include, beside the statistical component, the effect of all the possible systematics like EPID error positioning or deviation of the beam fluence shape from one exposition to the other.

Since the calibration matrix G is linked to the response of the EPID panel, it should not depend on the setup condition. We have thus compared calibration matrixes achieved with different phantom thickness, both with the same and with different energies (6 and 15 MV). To evaluate the agreement between different calibration setup, we have simply analyzed the distribution of the relative calibration matrixes differences.

#### C.3 Dose image

The following step was the dosimetric verification of the computed calibration coefficients. The calibration matrix G was thus applied to an image acquired with a square field of area, equivalent at the isocenter to $23\times 23\;{\text{cm}}^{2}$. This area was chosen to exclude the edges of the calibration array and we compared the resulting EPID dose image ($D_{\text{ij}}^{\text{EPID}}$, see Eq. (21)) to film measurements. For this comparison, we used a film located in a water‐equivalent slab phantom. Film measurement was performed using a 3 cm thick phantom, placed instead of EPID (for a SDD of 157 cm), with film inserted at 1.2 cm depth (3 cm phantom thickness and film depth of 1.2 cm were experimentally chosen in order to maximize the agreement between film and EPID dose image). Separately from the film irradiation, a measurement with EPID was performed.

Comparison consisted in the difference among the film dose image and the EPID dose image. To exclude the high‐gradient regions from the comparison process, the area taken in account was limited to $22.5\times 22.5\;{\text{cm}}^{2}$ equivalent at isocenter. The EPID pixel resolution was the real one ($0.4\times 0.4\;{\text{mm}}^{2}$).

The values of the calibration matrix G were readapted according to the resolution of the acquired images (0.4 mm) using a 2D linear interpolation method. As the PMS is computed over the low‐gradient dose region of the field, this process (the adaptation from the PMS pixels to the EPID pixels) leads to a negligible loss of information. Using a simple simulation procedure, the pixels with a resolution of 0.25 mm of a 23 cm width beam profiles where grouped to obtain 5 mm pixels and, finally, readapted to 0.25 mm through a linear interpolation. The maximum difference between the original and the recomputed profiles reaches the value of 0.1% (the length of analysis was limited to 22.5 cm). The original EPID resolution can thus be restored with an additional error of 0.1%.

We note that we used this setup to probe the correctness of the method by checking the reconstructed matrix against a precise reference, the film. The purpose of this comparison is, simply, the evaluation of the grade of correspondence between the dose image and the dose absorbed in a phantom, using a field covering the quasiwhole detector area.

The method for the in‐phantom dose reconstruction that we normally use is based on the back‐projection algorithm described by Wendling et al.^(19)^ In this particular case, the retro‐projection of the dose image was not performed in order to avoid data contamination from the convolution and deconvolution operations.

#### C.4 Film dosimetry

The 2D verification of the calibration results was performed through the comparison of the EPID dose images with radiographic films. As reference document for the film dosimetry we have chosen the report of the American Association of Physicists in Medicine (AAPM) Task Group 69.^(28)^

The films used for measurements were the EDR2 (Eastman Kodak Company, Rochester, NY), processed with a FP1500‐SAIEP film processor (SAIEP, Savona, Italy), and digitized with a VIDAR VXR 12‐bit scanner (VIDAR Systems/Contex Group, Stockholm, Sweden). Scanning was performed with a resolution of 75 dpi in both directions. Images were then corrected for background and smoothed to reduce the noise.

To determine the sensitometric curve, films were placed in a homogeneous slab phantom, with central axis of the beam perpendicular to the surface of the films. Thirteen dose points have been calibrated uniformly over the range from zero to 300 cGy. Dose delivered to the calibration films was independently measured with a Farmer‐type ionization chamber.

## III. RESULTS

### A. Single pixel gain deviation

As described in the section above, a single calibration process gives as result eight PSMs. For each pixel of the calibration matrix it is thus possible to compute an error, defined as standard deviation over the eight calibration coefficients. It is then possible to identify two components that contribute to the standard deviation: the first one is the statistical fluctuation of the single pixel count. The second one is due to the algorithm used for the PSM evaluation; departing from the reference pixel (chosen in the center of the sensor) the number of pixels involved in the calibration coefficients computation increases and, consequently, increases the global error due to the fluctuations of the counts.

**Figure 3:**
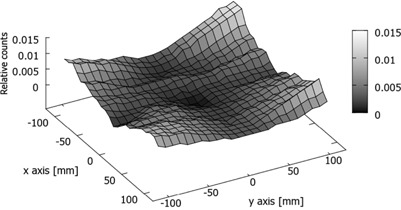
2D distribution of the standard deviation of pixel calibration coefficients. Measurements setup: 6 MV, 4 cm thick phantom.

Figure 3 shows the 2D distribution, over the detector area, of the standard deviations (computed over the eight PSMs) of the pixels calibration coefficients. Data were collected with

**Figure 4:**
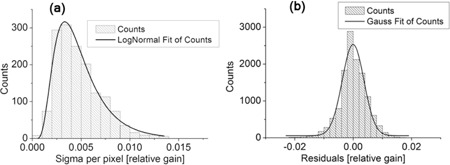
Distribution of standard deviation (sigma) (a) of pixels calibration coefficient; distribution of the residuals (b). Measurements setup: 6 MV, 4 cm thick phantom.

6 MV energy photons and intermediate scatter condition (4 cm thick phantom on the treatment couch). We observe that the deviation tend to increase both in the radial direction moving out from the EPID center and, mainly, departing from the axes of the detector. This confirms that the main contribution to the error comes from the number of pixels included in the computation. The same data plotted in Fig. 3 are collected in the histogram of Fig. 4(a). Data have been fitted assuming a log normal distribution. We determined a mean value equal to 0.4% and a standard deviation of 0.3%. As stated above, the maximum spread is located on the corners of the detector, reaching a value of 1.4%.

Moreover, the stability of the measurements in the single calibration process can be probed by studying the difference (defined “residual”) between the coefficient value of a pixel averaged over the eight calibration sets and each one of the single coefficient value. In Fig. 4(b) we show the distribution of the residuals, eight values for each pixel. The continuous line is the result of a Gaussian fit, with mean value zero and standard deviation 0.5%. Furthermore, all the values are contained within (‐2.5%: 2.5%). The analysis of the residuals distribution includes either the contribution of the statistical component of the error either the systematic ones, as for example due to the detector positioning error, and possible deviations of the fluence shape and magnitude.

### B. Energy and scatter dependence

**Figure 5:**
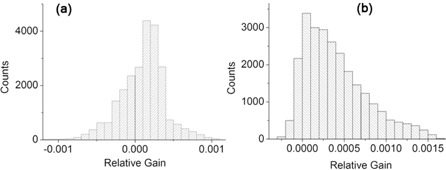
Differences of the calibration coefficients as a function of: (a) photon energies (6 MV versus 15 MV) and (b) scatter (20 cm thick phantom vs. no phantom).

To rule out the impact on the procedure of the photon energy and the front scatterer, the calibration coefficients have been determined with a photon energy both of 6 MV and 15 MV maintaining the same scatterer, and, in a second setup, fixing the energy and changing the scatterer. Figure 5(a) shows the distribution of the differences between the calibration matrix computed with 6 MV photon energy and the one obtained with 15 MV photon energy (both with 4 cm thick phantom over the couch). The mean value of the distribution is peaked at zero (99% Confidence Level) with the root mean square (rms) of the distribution equal to 0.03%. We thus infer that the two calibration matrixes are not significantly different.

In a similar way the dependence from the scatterer thickness has been evaluated. Two calibration matrixes have been computed with and without a full scatterer of 20 cm phantom. Figure 5(b) shows the differences between the two matrixes. Even if there is no statistical difference between the two populations (the mean value is zero with a confidence interval of 99%), the data distribution shows a clear asymmetry preferring a larger value when the scatterer is present. We interpret this asymmetry as due to the known overresponse of the EPID to scattered low‐energy photons that are more copious in the presence of the scatterer. The computed root mean square error of the distribution is 0.06%.

Analyzed data show that the dependence of the calibration procedure from the setup condition is much less than 1%. Nevertheless, to further reduce the uncertainty, an optimal calibration setup should be as similar as possible to the measurement conditions.

### C. EPID dose image

According to Eq. (21) (omitting the pixel indexes i and j), the EPID dose image is defined as $D^{\text{EPID}}={\text{IM}}^{\text{EPID}}\times G$, where ${\text{IM}}^{\text{EPID}}$ is the raw image acquired with EPID and G is the PMS calibration matrix.

To verify the dose image, a film was placed at the level of the EPID (see section above). Data were normalized to a central region of $1\times 1\;{\text{cm}}^{2}$.

**Figure 6:**
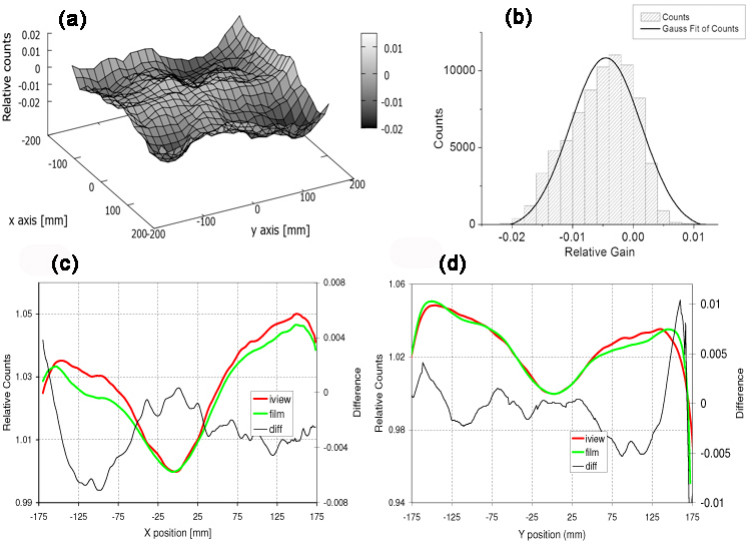
Comparison between film and EPID dose image for a square field of area equal to $23\times 23\;{\text{cm}}^{2}$ equivalent at the isocenter: (a) point‐to‐point differences in a 2D distribution, and (b) collected in a histogram; (c) cross‐plane and (d) inplane profiles of the same field determined with EPID (red line) and film (green line). The black line shows the difference between film and EPID.

Figure 6 shows the difference between film and EPID (relative) dose image. The largest differences (Fig. 6(a)) are located on the corners of the field, highlighting a slight overresponse of the EPID with respect to the film. Moreover, the distribution of the differences is reported in the histogram of Fig. 6(b). As a result of a Gaussian fit, the mean value of the distribution is −0.4% with a standard deviation of 0.6%. The difference between the mean value and zero, fixed a confidence level of 99%, is significant. For completeness, in Fig. 6(c) and 6(d) the cross‐plane and the in‐plane dose profiles for both detectors are presented.

**Figure 7:**
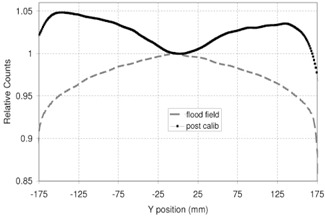
Flood‐field cross‐plane profile (dashed gray line) and post‐PMS calibration cross‐plane profile (black line with dots).

Finally, Fig. 7 shows the comparison between the in‐plane post‐PMS calibration profile (see Fig. 6(d)) and the relative flood‐field profile (before the PMS calibration).

### D. Reproducibility of the calibration results

Several effects can affect the reproducibility of the results: a) a variation of the beam output; b) a variation of the beam shape; and c) a deviation of the EPID shifting. In this subsection we consider the impact of the above effects.

Analyzing the beam output variation over a consistent number of irradiations with a fixed MU value, it was found the value of f to be in a range of ±0.2% and moreover to follow a normal distribution. Since this is a systematic error, its propagation over an iterative process of n steps is n times the error. A correction of this type has thus a paramount importance. With a simulation we studied the magnitude of the remaining error once the correction of Eq. (18) has been applied and we found that it becomes negligible.

The correction concerning the beam symmetry variation has been checked both with a simulation procedure and with measurements. We report the results related to the measurements. In this context, it was applied the PMSs evaluation, as described in the Material and Methods section 3.2 above, and the improvement in term of reproducibility was evaluated. Four different acquisitions were acquired in one day, and the relative PMSs were computed with and without the symmetry correction (in both cases the correction for the beam output variation was applied). Assuming the first one as reference, the comparison between the reference and the other PMSs were analyzed in terms of standard deviation of the distribution of the differences and in terms of maximum difference.

As shown in Tables 1 and 2, the standard deviation among the PMSs is less than 0.2%, being reduced by a factor in the range between 2 and 4 and, similarly, the maximum difference (located at the corners) is less than 0.8%, being a factor in the range between 2 and 4 smaller than before applying the correction.

Finally, the EPID shift error was evaluated. Projected to the isocenter plane and relative to a 5 mm shift, it was found to be within the range of −0.1mmto0.2mm. The error over the PMS computation was then evaluated, with a simulation procedure, in the case of an EPID shift error of 0.25 mm (the pixel dimension). The maximum sensitivity variation was about 0.4% and was located on the corners of the detector.

**Table 1:** Average and maximum difference among four different PMSs computed without the symmetry variation correction

*Not corrected*	*Acq 2*	*Acq 3*	*Acq 4*
RMS	0.7%	0.8%	0.4%
Abs (diff max)	1.8%	1.9%	1.5%

**Table 2:** Average and maximum difference among four different PMSs computed with the symmetry variation correction

*Corrected*	*Acq 2*	*Acq 3*	*Acq 4*
RMS	0.15%	0.16%	0.2%
Abs (diff max)	0.6%	0.5%	0.8%

## IV. DISCUSSIOM & COMCLUSIOM

A matrix of sensors can be exploited as a dosimetric tool if the response of each sensor to a fixed fluence is known. The calibration of a generic 2D detector can thus be defined like a matrix whose components are the relative gains of the measuring elements. In this paper we introduce an approach to calibrate the EPID, which is innovative with respect to the standard methods described in literature. The present method is based on a sequence of consecutive irradiations with constant fluence and beam shape.

The described algorithm produces as a result eight different matrixes of calibration. In the present analysis we used the average as the most‐like value. On the other hand, the computed standard deviation among the eight sets provides a direct assessment about the goodness of the whole procedure. We have observed that the standard deviation increases, both in radial direction from the center of EPID and with the increasing of the distance from the central axis (Fig. 3). The behavior is clearly an artifact of the algorithm. With our method, the calibration coefficient of a generic pixel is a function of the counts of the pixels up to the reference pixel along the same row or column: the greater the distance between the given pixel and the reference one (that we chose at the center of the EPID), the larger is the number of pixels that intervenes in the pixel computation and, consequently, the greater is the uncertainty. The mean value of the standard deviation is 0.4%, with a maximum spread located on the corners of the detector that reaches 1.4%.

A second, and similar, analysis is the distribution of the residuals: their standard deviation is 0. 5% and all the values are contained within −2.5% and 2.5%. Because the residuals take into account each single pixel determination with respect to the average value, a distribution with a skewness different from zero or a long side tail highlights the possible presence of an error in a single calibration process.

We studied also the dependence of the calibration from the photon energy and from the scatter radiation. Data collected show that the calibration setup affects the calibration coefficient of the pixels to a lesser extent than 1%, without a significant dependence from the photon energy. The quasicomplete independence of the PMS from the setup calibration conditions is the major advantage of this method with respect to the other experimental methods described in literature. Basically, most methods rely on an external dosimeter that is used to measure the beam shape (${\text{Image}}_{\text{ext}}$) which is then “imposed” to the EPID flood‐field (${\text{Image}}_{\text{FF}}$). Since the external dosimeter and the EPID respond in different way as a function of beam spectra, the ratio between the images ${\text{Image}}_{\text{ext}}$ and ${\text{Image}}_{\text{FF}}$ depend on the setup conditions: a difference in the calibration setup results in a difference in the PMS.

In the method presented in this paper, the independence from any external dosimeter reduces the uncertainty associated to the differences in the response as a function of the measurement conditions.

Once the calibration matrix was computed, the verification of the corresponding EPID (relative) dose image was performed through the comparison with film. Using a square field that covered the quasiwhole sensitive area, irradiations were done placing the film at the EPID position. The 2D distribution shows that the main differences are located on the corners of the field, highlighting a slight over response of EPID with respect to the film. This result (in agreement with the literature^6^, ^7^, ^8^, ^9^, ^10^) is then confirmed by the Gaussian fit of the distribution of the difference (see Fig. 6(b)): the mean value of the film versus EPID differences is −0.4% with an rms of 0.6%.

Finally, the reproducibility of the PMS was evaluated. Correcting the beam output and the beam symmetry variation among subsequent irradiations, the reproducibility was evaluated to be, on average, about 0.2%, with a pixel‐to‐pixel maximum discrepancy lower than 1%. Moreover, the uncertainty related to the positioning of the EPID at each step of the calibration has been determined to be smaller than 0.2 mm, which leads to a maximum sensitivity deviation smaller than 0.4%.

Summarizing the described method shows many advantages with respect to other described methods.

- The dependence from the setup conditions is almost negligible; however, this is not true if an external dosimeter is used as reference.
- As a result of the algorithm, eight set of PMSs are computed and the average values are used as the most‐like values. This leads to three main advantages. It reduces the error and allows the error evaluation. From the distribution of the errors related to each individual pixel, one can decide if the PMS is acceptable or not and, through the residuals analysis, allows to investigate if a particular error has occurred during the acquisition phase. Again, this is not possible if a standard method is used.
- The method is easy and fast to perform: it requires a sequence of five measurements which are then processed with a simple algorithm. Moreover, no dedicated images acquisition software is needed.

## Supporting information

Supplementary Material
