# Supplementary figures and images for: A new approach for the pixel map sensitivity (PMS) evaluation of an electronic portal imaging device (EPID)
Source: J Appl Clin Med Phys. 2013 Nov 4;14(6):234–50. doi: 10.1120/jacmp.v14i6.4420 (PMC5714628; doi:10.1120/jacmp.v14i6.4420)

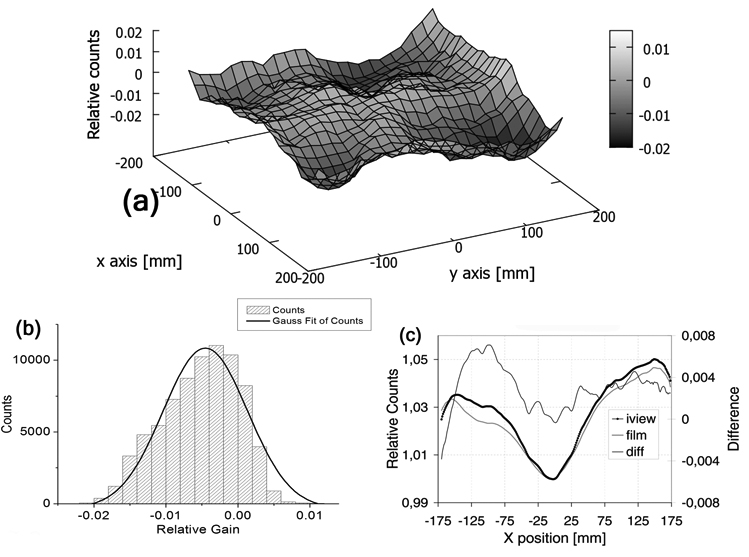

Supplement: Supplementary file 1 — Supplementary Material [file ACM2-14-234-s001.jpg]
